# Policy on Paper compared with Policy in Practice: A Study of Healthy Default Beverage Implementation and Enforcement in the United States

**DOI:** 10.1016/j.cdnut.2026.107674

**Published:** 2026-03-14

**Authors:** Samantha M Sundermeir, Megan R Winkler, Jennie N Davis, Alexandra Mack, Jennifer Falbe, Melissa Fuster

**Affiliations:** 1 Department of Epidemiology, Rollins School of Public Health, Emory University, Atlanta, GA, United States; 2 Department of Behavior, Social, and Health Education Sciences, Rollins School of Public Health, Emory University, Atlanta, GA, United States; 3 Institute for Global Nutrition, University of California, Davis, Davis, CA, United States; 4 Department of Social, Behavioral, and Population Sciences, Weatherhead School of Public Health and Tropical Medicine, Tulane University, New Orleans, LA, United States; 5 Department of Human Ecology, University of California, Davis, Davis, CA, United States

**Keywords:** public health nutrition, nutrition policy, health default beverage, policy implementation, policy enforcement, sugar-sweetened beverages

## Abstract

**Background:**

Healthy default beverage (HDB) policies, which require restaurants to offer healthier drinks with children’s meals, may reduce child sugar-sweetened beverage consumption. Although they are gaining popularity across the United States, restaurant awareness and compliance remains low, warranting deeper investigation into policy guidance and implementer knowledge.

**Objective:**

To examine implementing agencies’ awareness of HDB policies, the prevalence of implementation and enforcement practices and their alignment with policy documents, and challenges to implementation and enforcement.

**Methods:**

We used a mixed-methods approach combining an analysis of HDB policy documents and an online survey of implementers across jurisdictions with state or local policies. A codebook was developed to assess implementation and enforcement provisions in policy documents. Descriptive statistics were used to summarize survey-reported practices, and we examined alignment between these practices and the corresponding policy language. Nineteen policy documents (4 state, 15 local) and 64 survey responses from unique jurisdictions were analyzed.

**Results:**

Among jurisdictions, 46 (72%) were knowledgeable about their policy. Most state jurisdictions (86%) and all local jurisdictions reported using a communication strategy regardless of inclusion in policy documents. Restaurant compliance was typically assessed through in-person restaurant visits (state: 78%; local: 42%). Equity-related implementation considerations included additional time for policy implementation (state: 43%; local: 60%) and technical support (state: 72%; local: 60%). Most state (67%) and local (73%) jurisdictions reported issuing warnings and fines for enforcement. Equity considerations for enforcement were reported for some state jurisdictions (27%) and included additional time to become compliant (75%) and issuing warnings without escalating to fines (25%). Implementation misalignment between practices and documents often reflected jurisdictions exceeding policy documents, whereas enforcement misalignment between practices and documents often involved inconsistent or incorrect use of the enforcement strategy in the policy document and misalignment of reported funding allocation.

**Conclusions:**

Although jurisdictions frequently expanded beyond written implementation requirements, enforcement knowledge and practices inconsistent with policy documents highlight potential gaps in resources, training, and infrastructure. Efforts to bolster implementer capacity and promote equitable approaches may increase the ability of localities to implement and enforce HDB policies.

## Introduction

About 70% of children aged 2–13 y exceed recommendations for added sugar intake, with sugar-sweetened beverage (SSB) consumption being the top source of added sugar [1,2]. Policy and environmental changes are needed to help reduce excessive added sugar consumption given the associated negative health outcomes for children, such as unhealthy weight gain, and prolonged intake poses an increased risk of dental caries, type 2 diabetes, and heart disease later in a child’s life [3,4]. One avenue increasingly being explored are policies designed to reduce SSB consumption [5].

Healthy default beverage (HDB) policies have the potential to reduce child SSB intake in restaurants by requiring restaurants to only offer healthier drinks (e.g., water, milk, and small-sized 100% juice) as the default beverage option with children’s meals [5]. HDB policies may make selecting healthier beverages the “easy” choice, and are gaining traction in the United States [6,7]. Previous research indicates that consuming restaurant meals, especially children’s meals, is associated with higher SSB intake [8]. As such, the use of HDB policies in restaurants may be a promising venue to target child SSB consumption as 62% of children and adolescents consume meals from restaurants on a given day [9].

Since 2019, 28 local jurisdictions and 4 states in the United States have enacted and implemented HDB policies [10]. To our knowledge, published research examining HDB policies have yet to examine effectiveness of the policies to reduce SSB consumption beyond one simulation study [11]. Emergent research has focused on awareness and compliance, showing that policy awareness among restaurants is low and consequently compliance is also low [[12], [13], [14], [15], [16], [17], [18], [19]]. For example, a study exploring policy awareness among restaurant managers in California and Wilmington, Delaware found that 29.3% and 0% of managers were aware of the policy in their respective locations [18]. That study also showed overall low compliance when comparing menus/menu boards before and after policy enactment [15,18], as the number of menus/menu boards listing only policy-consistent beverages significantly increased in California but remained unchanged in Delaware 7 to 12 months after policy enactment [15,18]. Other studies examining HDB policy adherence for online menus found that websites and online food ordering platforms were not offering children’s meal beverages consistent with state HDB laws, especially in low-income neighborhoods [13,17,19].

These initial findings demonstrate the need to take a closer look at policy implementation and enforcement language within the HDB policies themselves as well as on-the-ground practices being deployed by local implementing agencies. Both state and local level HDB policies are typically implemented and enforced by local health departments. However, the specific division, unit, or personnel responsible (e.g., environmental health, nutrition, and chronic disease prevention staff) varies widely by jurisdiction. Similarly, the procedures and mechanisms for implementation and enforcement, such as the communication strategy used to inform restaurants about the policy, incorporation into routine restaurant inspections, complaint-driven investigations, and formal citation processes, are determined locally based on existing regulatory structures and administrative capacity. For example, a previous study of healthy kids’ meal policies (which include HDBs) found variation across jurisdictions in how the laws are written and structured [6]. Policies provided limited detail on implementation processes, oversight authority, penalties, or responsible agencies, leaving important operational decisions to local discretion. These administrative differences may play a key role in bridging the gap between policy and practice; understanding how policy documents may translate to on-the-ground practice is essential for strengthening HDB policies and their public health impact [20].

Therefore, we aimed to answer the following research questions: *1*) Do implementing agencies in jurisdictions with HDB policies know about their current local or state policy? *2*) Among those who are aware of the policy, how prevalent are practices related to implementation and enforcement, and how aligned are they with their current HDB policy? *3*) What are the challenges related to implementation and enforcement of HDB policies? This information is a needed addition to move beyond assessing awareness among restaurants and identifying barriers and facilitators to effective policy implementation.

## Methods

We used a convergent parallel analysis [21], combining an online survey of HDB policy implementers (e.g., local health department staff who implement and enforce HDB policies) and HDB policy document analysis.

### Survey design

We developed a survey to distribute to HDB policy implementers to ask specific questions about policy knowledge and implementation and enforcement practices. Survey questions were developed iteratively by the research team based on gaps identified in existing HDB policy literature [6,12], group discussions, review of an existing survey of nutrition policy implementers in the United Kingdom [22], and initial review of the policy documents. The survey was piloted by 2 respondents from a local jurisdiction with an HDB policy, and revisions were made based on their feedback. The final survey had 6 sections including general information, policy development, policy implementation, and policy enforcement. The survey was built and distributed online using Qualtrics survey software.

### Survey sampling strategy

We aimed to survey every United States jurisdiction implementing an HDB policy at the state or local level. At the time (September 2024), 4 states had state-wide HDB policies (California, Delaware, Hawaii, and Illinois), and 18 jurisdictions had policies at the local level (e.g., city, county). In some cases, local policies existed prior to the enactment of a statewide policy. In those cases, we used the state policy document given that the state law was the minimum expectation for all jurisdictions across the state. Given that local health departments are the main implementing agency for state and local HDB policies [6,10], and that there is no systematic way to identify them all, we opted to search the National Association of County and City Health Officials website [23] and exported contact information for all local health departments from each locality. For state-level policies, we identified a census of 149 city/county health departments: 57 in California, 2 in Delaware, 4 in Hawaii, and 86 in Illinois. We contacted health department staff in all 167 state and local jurisdictions via email between 22 January and 30 April, 2025, and asked them to complete the survey within 2 wk. If there was no response, follow-up emails were sent every 2 wk for ≤7 points of contact. Phone calls were made to 3 jurisdictions for which email addresses were unavailable or inactive. To ensure we reached the most knowledgeable contact, participants had the option to provide alternative contacts with additional policy and enforcement knowledge at multiple points throughout the survey or in response to our email.

### Policy document collection

HDB policy documents (enacted HDB policies from state legislation, municipal codes, etc.) were first drawn from an existing inventory of United States local, state, and Tribal SSB policies enacted prior to 31 May, 2023 compiled by Davis et al. [24]. Documents from this database were included if the policy text referenced HDBs in restaurant children’s meals. We then updated the database according to the methodology of Davis et al. [24] by systemically searching policy databases (American Legal Publishing Code Library, Municode, NexisUni, and Westlaw) and searching Center for Science in the Public Interest’s website for additional HDB policies enacted through 30 September, 2024.

### Ethical considerations

Individual names and work contact information (e-mail, phone number) were collected to identify potential participants via publicly available records. No other personal or identifying information was collected other than participants’ state, city, or county of employment. The study was deemed exempt by the Institutional Review Board at Tulane University School of Public Health. Participants were offered a $25 electronic Visa gift card for participation.

### Analysis

We conducted a convergent parallel analysis in which the survey and policy document review were implemented at the same phase of the project, analyzed separately and joined for interpretation [21]. Descriptive statistics were used to summarize each section of the survey. We then generated frequencies to examine the number of jurisdictions that reported on the implementation and enforcement components of interest coded in the policy documents.

A codebook for HDB policy characteristics was developed based on a codebook previously published by Perez et al. [6]. We updated and expanded the codebook to address our research questions by adding codes from the SSB inventory analysis by Davis et al. [24] and through team discussions. General policy characteristics codes included jurisdiction, policy effective date, policy sponsor and party, and policy purpose, as well as specific information about implementation and enforcement. Implementation codes included instructions for communicating the policy to restaurants/community, how to assess restaurant compliance, and equity considerations. Equity was defined as any reference within a policy’s implementation or enforcement sections that prioritized the health and well-being of groups historically marginalized, underrepresented, and/or under-resourced [25]. Additional aspects of were also coded based on a definition adapted from the Perez et al. research tool [6]: language that referenced differences between population groups, such as gender, race or ethnicity, health status, education, income, disability, geographic location, or sexual orientation, and/or unjust differences (inequities) due to systemic social conditions. For implementation and enforcement, these definitions were operationalized as mention of considerations for small and/or locally owned restaurants that might have limited resources, funds, and staff such as additional time for implementation. Enforcement codes included penalties for noncompliance (i.e., warnings, monetary fines, and escalating fines), funding to support enforcement activities, and equity considerations for small and/or locally owned restaurants such as additional technical support or modified/reduced penalty schedules. The full version of the codebook, including code definitions, has been published elsewhere [10]. Descriptive statistics were used to summarize the coded policy characteristics and the number of policy documents that included each policy component overall and by state or local jurisdiction. For this analysis, we only included policy documents with a corresponding survey response (*n* = 4 state and *n* = 15 local documents).

Next, we merged the 2 data sources to examine the alignment (or misalignment) of implementation and enforcement practices reported by jurisdictions in the surveys with their corresponding policy document. Reported implementation and enforcement practices were considered “aligned” if they matched the policy document (either with the absence or presence of certain information). Reported implementation and enforcement practices were considered “misaligned” if they did not match what is written in the policy document. Whether the practices and documents matched was determined on a case by case basis; although they did not have to match verbatim, similar key words/terms and activities had to be named in order to match (e.g., communication strategies used terms such as flyers, emails, website, or enforcement activities listed specific warning/fine schedules and fine amounts). Among misaligned practices, some were “positively misaligned,” meaning that jurisdictions went above and beyond what was written in their policy document in a way that provided more detail or reported additional considerations. Others were “negatively misaligned” meaning they reported less detail or were not doing the activities outlined in the policy. Alignment of implementation and enforcement strategies were grouped into the following categories: communication strategy, compliance assessment instructions, implementation equity considerations, enforcement strategy, enforcement funding, and enforcement equity considerations. We also separately assessed survey respondents’ self-reported confidence in conducting enforcement activities, such as identifying which restaurants were subject to the policy, identifying noncompliance, and administering notices/fines for noncompliance. Responses ranged from 1 (not confident at all) to 4 (very confident), and the mean responses are reported.

Finally, we analyzed a small number of responses to an open-ended question where respondents could describe challenges related to policy implementation and enforcement.

## Results

### Response rate

**FIGURE 1:**
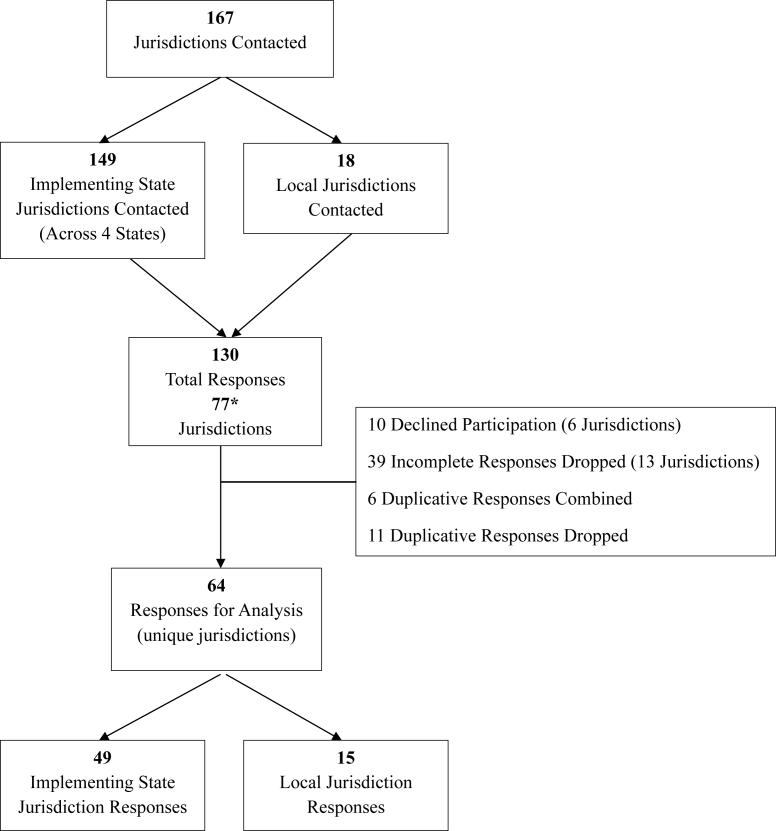
Healthy default beverage implementer survey responses flow diagram. ∗The total number of unique jurisdictions who responded to the survey in the first instance is unknown as many who declined or did not fully complete the survey did not enter their jurisdiction.

We received survey responses from 77 jurisdictions of the 167 contacted (46% response rate); however, this is likely an underestimate as the total number of unique jurisdictions who responded in the first instance is unknown as 31 respondents who declined or did not fully complete the survey did not disclose their jurisdiction (Figure 1). Confirmed state and local response rates were as follows: California, 42% (24/57); Delaware 50% (1/2); Hawaii 25% (1/4); Illinois 40% (34/86); city/county (local) 90% (16/18).

A total of 130 individual survey responses were received, including multiple responses from individual jurisdictions (Figure 1). Of those, responses were excluded from those who declined consent to participate in the full survey (*n* = 10, 4 jurisdictions) and from incomplete surveys (*n* = 39 responses, 13 jurisdictions). For jurisdictions that included >1 response, we merged these by either combining answers from incomplete surveys to create 1 complete survey response (when respondents completed complimentary survey sections) (*n* = 6), or the most complete response per jurisdiction was retained and the other(s) dropped (*n* = 11). This left a final analytic sample of 64 responses from unique jurisdictions.

### Policy knowledge

Of the 64 jurisdictions, 18 only answered the “general information” section of the survey and were therefore categorized as “not knowledgeable” about the policy. Of note, 1 local and 2 state jurisdictions reported they knew of the policy but did not have the time or capacity for implementation and enforcement or did not have enforcement authority; they were categorized as “not knowledgeable” for the purpose of this analysis because they did not complete any of the substantive portions of the survey. Most of the jurisdictions that were not knowledgeable were from localities with a state-wide policy (Illinois: *n* = 9, 50%; California: *n* = 8, 44%) and had policies that went into effect several years prior to the survey (effect date: 2019–2021), compared to more knowledgeable jurisdictions where the policy enactment year ranged from 2019 to 2025 (Table 1). Of the jurisdictions that were knowledgeable about the policy (*n* = 46, 72%), completeness across and within each section of the survey varied, and therefore sample sizes change throughout the results (see Supplemental Figure 1 and table footnotes).

**TABLE 1:** Characteristics of the jurisdictions who were and were not knowledgeable about their healthy default beverage policy.

Characteristic *n (column %)*	Overall *n* = 64	Not knowledgeable about policy *n* = 18	Knowledgeable about policy *n* = 46
State-level policies			
Illinois	26 (40.6)	9 (50.0)	17 (37.0)
California	21 (32.8)	8 (44.4)	13 (28.3)
Delaware	1 (1.6)	0	1 (2.2)
Hawaii	1 (1.6)	0	1 (2.2)
Local-level policies	15 (23.4)	1 (5.6)	14 (30.4)
Denver, CO Golden, CO Lafayette, CO Longmont, CO Louisville City, CO New Orleans, LA Montgomery County, MD Charles County, MD St. Louis, MO New York City, NY Cleveland, OH Columbus, OH Toledo, OH Youngstown, OH Philadelphia, PA			
Jurisdiction level			
State	49 (76.6)	17 (94.4)	32 (69.6)
Local	15 (23.4)	1 (5.6)	14 (30.4)
Jurisdiction size			
Median	103,984	77,665	109,650^1^
IQR	27,232-714,336	21,836-714,336	30,439- 439,035
Policy effective year			
2019	21 (32.8)	8 (44.4)	13 (28.3)
2020	4 (6.3)	1 (5.6)	3 (6.5)
2021	29 (45.3)	9 (50.0)	20 (43.5)
2022	4 (6.3)	0	4 (8.7)
2023	2 (3.1)	0	2 (4.3)
2024	1 (1.6)	0	1 (2.2)
2025	3 (4.7)	0	3 (6.5)

1 Denominator varies for this item due to missing. *n* = 45 as one jurisdiction in California did not list their exact city/county and therefore the population size is unknown.

### Overall reported implementation and enforcement practices

Overall, most jurisdictions with either a state (*n* = 18, 86%) or local (*n* =13, 100%) level policy reported using a communication strategy (Table 2). Restaurant compliance was typically assessed through in-person restaurant visits (state: *n* = 14, 78%; local: *n* = 5, 42%) rather than self-certification. Equity-related implementation considerations included additional time for policy implementation (state: *n* = 3, 43%; local: *n* = 3, 60%) and technical support (state: *n* = 5, 72%; local: *n* = 3, 60%) for small or locally owned restaurants. Most state (*n* = 10, 67%) and local (*n* = 8, 73%) jurisdictions reported issuing warnings and fines for enforcement. Equity considerations for enforcement were only reported for 4 state jurisdictions (27%) and included additional time to become compliant (*n* = 3, 75%) and issuing warnings without escalating to fines (*n* = 1, 25%).

**TABLE 2:** Healthy default beverage implementation and enforcement practices reported by jurisdiction level.

	Jurisdictions with state HDB policy	Jurisdictions with local HDB policy
Implementation, *n* (%)	*n* = 21	*n* = 13

Reports using a communication strategy	18 (85.7)	13 (100)
Reports details about assessing restaurant compliance	18 (85.7)	12 (92.3)
Strategies used^1^		
Existing licensing database	4 (22.2)	6 (50)
Restaurant self-certification	2 (11.1)	1 (8.3)
In-person restaurant visits	14 (77.8)	5 (41.7)
Menu submission	4 (22.2)	1 (8.3)
Other	2 (11.1)	1 (8.3)
Reports equity considerations for implementation	7 (33.3)	5 (38.5)
Considerations^1^		
Additional time for implementation	3 (42.9)	3 (60)
Additional technical support	5 (71.4)	3 (60)
Additional financial support	1 (14.3)	3 (60)

Enforcement, *n* (%)	*n* = 15	*n* = 11

Reports using an enforcement strategy (warnings, fines)	10 (66.7)	8 (72.7)
Reports details about enforcement funding	14 (93.3)	11 (100)
Reports equity considerations for enforcement	4 (26.7)	0
Considerations^1^		
Additional time to become compliant	3 (75)	0
Warnings only—do not issue fines	1 (25)	0

Abbreviation: HDB, healthy default beverage.

1 For these questions, respondents could “select all” that applied, so data may not total to 100%.

### Alignment of implementation practices with policy documents

**FIGURE 2:**
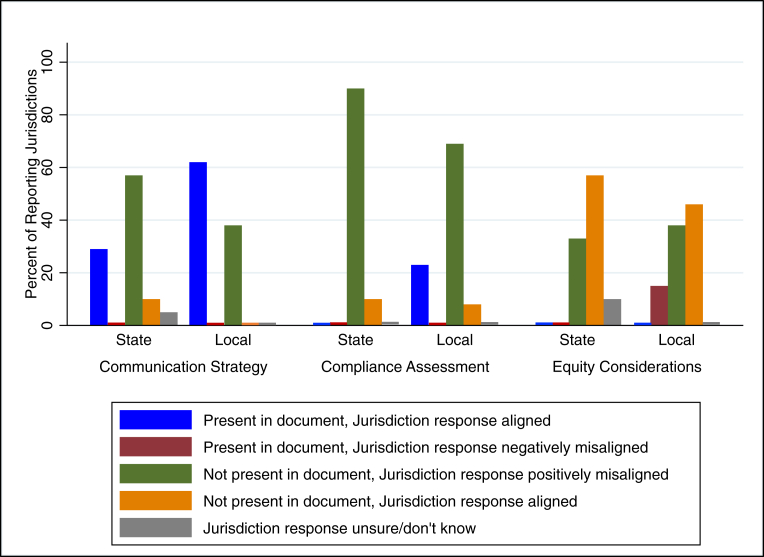
Alignment classification of implementation practices with policy documents by Jurisdiction Level [State: *n* = 21 (*n* = 20 for compliance assessment); Local *n* = 13]

We now turn to whether jurisdictions’ reported activities aligned with their specific written policy. Implementation details of interest for this analysis were the alignment on how to communicate the policy to the public and restaurants (e.g., print materials, providing technical assistance, and considering different languages), how to assess restaurant compliance, and equity considerations. Figure 2 presents the alignment classifications of implementation practices reported by jurisdictions with their corresponding policy document, organized by jurisdiction level (state or local). Supplemental Table 1 provides frequencies used to generate Figure 2.

#### State implementation

Of the 4 states with state-level HDB policies, 21 implementing jurisdictions answered implementation questions. Fourteen jurisdictions were from a state in which the policy documents did not specify a communication strategy for disseminating the policy requirements to restaurants and/or communities, but more than half of respondents (*n* = 12, 57%) reported using a communication strategy anyway, going above and beyond the written document (e.g., distributing flyers, sending emails, in-person visits, and websites about the policy). This was an example of a positive misalignment (Figure 2).

None of the state policy documents included specific details about initial compliance assessment (e.g., restaurants must submit a self-certification form to confirm compliance, or restaurants must submit their menu for approval). However, in practice, most jurisdictions (*n* = 18, 90%; total *n* = 20 for this question) reported using in-person visits/inspections, menu submissions, or self-certification to assess compliance, again highlighting positive misalignment (Figure 2).

Similarly, none of the state policy documents included equity considerations. In practice, 7 (33%) jurisdictions reported using special considerations for small and/or locally owned restaurants, including providing additional technical support or additional time for implementation (i.e., positive misalignment) (Figure 2).

#### Local implementation

There were 13 jurisdictions with a local HDB policy that answered implementation questions. Among those, 8 (62%) were from jurisdictions with a policy document that specified a communication strategy, and all reported using a communication strategy that aligned with their policy document (Figure 2). Five (39%) jurisdictions had local policy documents that did not include a communication strategy, yet all reported using a communication strategy anyway (i.e., positive misalignment). Three (23%) local jurisdictions had policy documents that included specific details about initial compliance assessment, and all 3 jurisdictions reported these details in the survey, aligning with the policy. Most local jurisdictions (*n* = 10, 77%) did not have documents that included this information, yet 9 (69%) still reported positive misalignment by using a system (self-certification, menu review, in-person visits) to confirm compliance.

Two (15%) local policy documents included equity considerations in their implementation language, with both requiring educational/informational outreach about the policy in multiple languages including Spanish. However, neither of the survey responses from those 2 jurisdictions reported those considerations (i.e., negative misalignment). Among the 11 jurisdictions without equity language in their policy documents, 5 (46%) reported equity considerations for implementation, such as creating materials in multiple languages, additional time for implementation, and additional technical and financial support (i.e., positive misalignment) (Figure 2).

### Alignment of enforcement practices with policy documents

**FIGURE 3:**
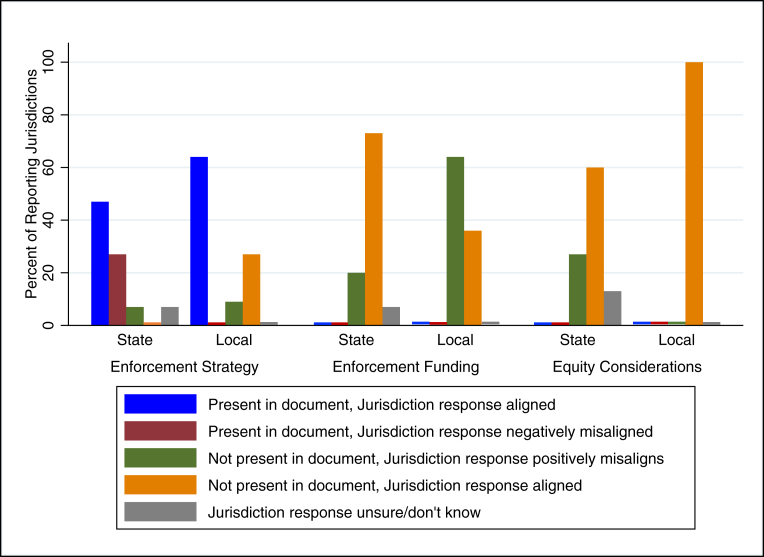
Alignment classification of enforcement practices with policy documents by jurisdiction level. State: *n* = 15 (*n* = 13 for enforcement strategy); Local: *n* = 11.

Enforcement details of interest for this analysis included information such as the enforcement strategy (schedule for issuing warnings and fines), funding for enforcement, and equity considerations. Figure 3 presents the alignment (or misalignment) of enforcement practices reported by jurisdictions with their corresponding policy document, organized by jurisdiction level (state or local). Supplemental Table 2 provides frequencies used to generate Figure 3.

#### State enforcement

Of the 4 states with state-level HDB policies, 15 implementing jurisdictions answered questions about enforcement. Three state documents (California, Delaware, and Illinois) outlined enforcement details such as a schedule for notices/fines or stated that there should be no fines associated with the policy. Of the jurisdictions from states with a state-level policy, 12 (92%; denominator is 13 for this question) had a state-level policy that outlined enforcement details. Of those, 7 (47%) reported enforcement details that aligned with their corresponding policy document, while 4 (27%) either reported not using an enforcement strategy (i.e., negative misalignment), reported details that did not align with their policy document, or did not know (Figure 3). For example, 2 jurisdictions reported a schedule and increments for notices/fines that did not align with their policy document (i.e., negative misalignment). There was 1 (7%) jurisdiction from a state in which the policy document did not outline enforcement details, yet they reported using specific enforcement approaches, including providing written notices for noncompliance (i.e., positive misalignment).

None of the state documents described funding sources for enforcement, which was in alignment with survey responses from 11 (73%) jurisdictions (Figure 3). However, 3 (20%) reported receiving some form of funding from a government source (i.e., positive misalignment).

None of the state documents included equity considerations in enforcement language. However, 4 (27%) jurisdictions reported using special considerations for enforcement at small and/or locally owned restaurants (i.e., positive misalignment); all 4 reported allowing additional time to become compliant before issuing a formal notice/fine. Two (13%) jurisdictions reported they were not sure if there were any equity considerations for enforcement.

#### Local enforcement

There were 11 jurisdictions with a local policy about which respondents answered questions about enforcement. Of those jurisdictions, 7 (64%) had a local policy document that outlined enforcement details, and all 7 respondents correctly reported enforcement details that aligned with their corresponding policy document. Among the 4 (36%) jurisdictions that had local policies that did not include enforcement details, 1 (9%) reported using an enforcement strategy (e.g., notices, fines; representing positive misalignment).

As in the case of state-level policies, none of the local policy documents described funding sources for enforcement activities. In alignment with the policy documents, 4 (36%) jurisdictions reported not receiving funding for enforcement, while 7 (64%) reported receiving some form of funding from the government or another source (positive misalignment). Similarly, none of the local policy documents included equity considerations in enforcement language. In alignment with this, none of the local jurisdictions reported using an equity enforcement strategy.

### Challenges and confidence with implementation and enforcement

In open text responses (*n* = 20: 11 state, 9 local), jurisdictions with state and local policies reported similar policy implementation and enforcement challenges. Although a few jurisdictions with newer policies reported smooth implementation thus far, other challenges included staff capacity limitations, lack of funding, lack of guidance from the state, and lack of restaurant awareness (Supplemental Table 3).

When asked about confidence in conducting enforcement activities in jurisdictions with warnings/fines schedules in place, both state and local jurisdictions were less confident in administering notices/fines [averaging 2.9 ± 0.9 of 4 (*n* = 7) and 2.7 ± 1.0 of 4 (*n* = 6), respectively] compared to other enforcement activities, such as identifying which restaurants were subject to the policy (Table 3).

**TABLE 3:** Confidence^1^ in conducting healthy default beverage policy enforcement activities by jurisdiction level.

	Jurisdictions with state HDB policy (*n* = 15)	Jurisdictions with local HDB policy (*n* = 11)
	mean (SD)	
Confidence in identifying which restaurants are subject to the policy	3.3 (1.1)	3.7 (0.6)
Confidence in identifying when a restaurant is not in compliance	3.1 (1.1)	3.3 (1.0)
Confidence in providing technical assistance to restaurants	3.0 (1.1)	3.1 (1.0)
Confidence in providing technical assistance to inspectors	2.9 (1.1)	3.1 (1.0)
Confidence in administering notices/fines	2.9^2^ (0.9)	2.7^2^ (1.0)

Abbreviation: HDB, healthy default beverage.

1 Responses ranged from 1 (not confident at all) to 4 (very confident).

2 Denominator varies for this item as we restricted to only jurisdictions with a policy document that requires fines and who reported those fines correctly (*n* = 7 state jurisdictions and *n* = 6 local jurisdictions).

## Discussion

This study explored the implementation and enforcement of HDB policies across jurisdictions with either local- or state-level policies using a mixed-methods approach. Our findings suggest that although awareness of the HDB policies among implementers was relatively high, particularly in jurisdictions with local policies, there was positive misalignment among implementation activities, meaning that implementers went above and beyond what was required in the policy document. However, the opposite was found among enforcement activities, where negative misalignment was found, especially for state-level HBD policies, denoting uncertainty around issuing warnings and fines and funding source. These gaps may reflect a disconnect between policy development and actionable guidance for successful implementation. This could have a trickle-down effect on restaurant managers and explain why prior evaluations of HDB policies have shown low restaurant-level awareness and compliance [12,13,15,17,18]. Previous research found that restaurant staff were largely supportive of HBD policies but were concerned about implementation logistics (e.g., staff training), weak enforcement mechanisms, and lack of community education on the topic [12,26]. Strengthening implementation and enforcement knowledge and infrastructure could bridge this gap and lead to a more effective policy.

Despite these misalignments, some jurisdictions demonstrated proactive behavior, especially for implementation activities, extending their efforts beyond what was required in their policy documents. For example, certain implementers who engaged in activities such as direct communication with restaurants reported clear approaches for initial compliance checks (e.g., in-person visits, menu submission) and, in some cases, made explicit efforts to incorporate equity considerations, such as additional time or technical support, into their implementation approach. These activities suggest that local organizational capacity can play a key role in whether a policy is effectively implemented.

Our study results point to the need of addressing enforcement given the lack of reported use of warnings and fines. Contrary to other SSB reduction policies like excise taxes, HDB policy enforcement requires on-the-ground monitoring and enforcement by local agencies to continuously review menus for compliance. Also, unlike taxes, this policy does not generate revenue, further limiting funding for enforcement activities and potentially enforcement motivation, given competing priorities. Nevertheless, other policies that also require on-the-ground monitoring, such as healthy checkout ordinances and menu calorie labeling, appear to have elicited higher compliance than HDB policies [27,28], and future research should identify reasons for these differences in compliance. However, we did find that many HDB policies did not clearly designate an enforcement strategy, and respondents from several jurisdictions did not accurately describe their enforcement protocol. In the challenges to implementation and enforcement open text responses, one jurisdiction suggested building compliance checks into food inspection reports; however, this would also require additional training or guidance on allowable beverages for inspectors to make quick assessments, following similar protocols used to assess menu-labeling compliance [29]. Moreover, jurisdictions vary in how restaurant inspections are carried out and the level at which inspections are conducted. For example, a local HDB policy with a state-level restaurant inspection procedure may face additional difficulties for enforcement and cross-agency communications. Moving beyond complaint-based enforcement toward more proactive, well-funded strategies with clear training on the policy’s nutritional standards could enhance the impact of these policies.

Several key challenges with implementation and enforcement were apparent across jurisdictions. The most frequently cited barriers to effective implementation and enforcement were a lack of staff capacity, insufficient funding, and lack of knowledge around nutritional requirements. This aligns with existing nutrition policy implementation literature, which underscores the importance of organizational readiness/capacity, clear guidance and training, and integration into existing systems [[30], [31], [32], [33]]. Collectively, these findings suggest that the success of HDB policies hinges not only on adoption but also on the availability of clear, well-resourced implementation and enforcement strategies, implying the need for policies to be well-funded from the start. Governments considering the adoption of similar laws should prioritize clear nutrition definitions and funding for enforcement activities.

Our study also documents lack of awareness among some policy implementers, which appears to be associated with local jurisdictions implementing state-level policies and older policies. The difference by jurisdiction level (state compared with local) has implications as these policies scale up from cities and counties to state levels. This scaling up is important for making a public health impact, yet it must be done in a manner that ensures information is shared across key players to ensure adequate adoption. This is especially true for state level policies that have been successfully scaled to encompass an entire state but need to be sufficiently translated to each local implementing agency across the state. Differences in policy enactment date may demonstrate that policy commitment wanes over time as competing priorities arise or institutional knowledge is lost with staff changeover. One opportunity to enhance policy implementation could be putting systems in place to automate policy notification to certify/recertify restaurant compliance, frequency of menu collection and self-certification for ongoing compliance review, along with other automatic notification such as reminders for annual collection of tax forms.

This study showcases key strengths, including our systematic sampling approach and our mixed-methods study design, allowing us to assess implementation and enforcement factors through 2 data sources: policy documents and the experiences of implementers collected via the survey. Limitations include self-reported data, which was dependent on individual knowledge. This may have resulted in limited sample sizes for some of the survey responses described above. Although we engaged in a systematic outreach process, resulting in a reasonable response rate (46%), it is still possible that we did not reach the best contact person for the survey or that the contact did not have the time or capacity to complete the survey. We also cannot assess whether alignment classifications translate into actual policy implementation outcomes, as such analysis is beyond the scope of this research; however, future studies may examine associations between alignment, implementation outcomes, and policy outcomes as additional data become available through formal policy evaluations.

In conclusion, this study explored the knowledge and practices of HDB policy implementers and enforcers at state and local levels and their alignment with policy documents. We found that although HDB policy awareness was relatively high among policy implementers, there were gaps in enforcement knowledge and practices, particularly among jurisdictions with a state-level policy. Understanding barriers and facilitators to effective policy implementation and enforcement is essential for strengthening jurisdictions’ capacity and support infrastructure and may increase the ability of localities to implement and enforce HDB policies.

## Author contributions

The authors’ responsibilities were as follows – SMS, MRW, JND, JF, MF: designed the research; MF, MRW, JF: obtained funding; SMS, JND, AM: conducted the research; SMS: analyzed the data; SMS, AM, MF: wrote the paper; SMS, MF: had primary responsibility for final content; and all authors: read and approved the final manuscript.

## Data availability

Data described in the manuscript, code book, and analytic code will be made available upon request pending application and approval.

## Funding

This research was funded by Healthy Eating Research, a Program of the Robert Wood Johnson Foundation. SMS is supported by a National Heart, Lung, and Blood Institute training grant (T32HL130025). JF was supported by the USDA/National Institute of Food and Agriculture Hatch project 7005204. The content is solely the responsibility of the authors and does not necessarily represent the official views of the National Institutes of Health or the USDA. Funding agencies had no role in the design, analysis or writing of this article.

## Declaration of generative AI and AI-assisted technologies in the writing process

The author(s) declare that no generative AI or AI-assisted technologies were used in the writing of this manuscript.

## Conflict of interest

The authors report no conflicts of interest.
