# Supplementary material for: Policy on Paper compared with Policy in Practice: A Study of Healthy Default Beverage Implementation and Enforcement in the United States
Source: Curr Dev Nutr. 2026 Mar 14;10(4):107674. doi: 10.1016/j.cdnut.2026.107674 (PMC13088997; doi:10.1016/j.cdnut.2026.107674)
Supplement: Multimedia component 1 [file mmc1.docx]

**Supplemental Figure 1:** Healthy Default Beverage Implementer Survey Completeness Flow Diagram

Jurisdictions responded to survey

(n=64)

Jurisdictions knowledgeable about the survey

(n=45)

Jurisdictions knowledgeable with state HDB (n=31)

Jurisdictions knowledgeable with Local HDB (n=14)

Jurisdictions knowledgeable about implementation (n=21)

Jurisdictions knowledgeable about enforcement (n=15)

Jurisdictions knowledgeable about implementation (n=13)

Jurisdictions knowledgeable about enforcement (n=11)

**Supplemental Table 1:** Alignment classification of implementation practices with policy document by jurisdiction level

|  | **Jurisdictions with State HDB Policy**  **(N=21)** | **Jurisdictions with Local HDB Policy**  **(N= 13)** |
| --- | --- | --- |
| **Communication Strategies** | **N (%)** | **N (%)** |
| *Policy document specifies a communication strategy* | N= 7 | N=8 (61.5) |
| Reports using a communications strategy (Aligns)  Reports not using a communication strategy (Negatively Misaligns)  Don’t know | 6 (85.7)  0  1 (14.3) | 8 (100)  0 |
| *Policy document does NOT specify a communication strategy* | N=14 | N=5 (38.5) |
| Reports NOT using a communications strategy (Aligns)  Reports using a communication strategy (Positively Misaligns) | 2 (14.3)  12 (85.7) | 0  5 (100) |
| **Compliance Assessment Instructions^a^** | **N (%)** | **N (%)** |
| *Policy document includes instructions about how to assess compliance (e.g., self-certification, menus submitted for approval)* | N= 0 | N=3 (23.1) |
| Reports using the named strategy for compliance assessment (Aligns)  Reports NOT using a strategy for compliance assessment (Negatively Misaligns) | -  - | 3 (100)  0 |
| *Policy document does NOT include instructions about how to assess compliance (e.g., self-certification, menus submitted for approval)* | N= 20 (100) | N=10 (76.9) |
| Reports NOT using a strategy for compliance assessment (Aligns)  Reports using a strategy for compliance assessment (Positively Misaligns) | 2 (10)  18 (90) | 1 (10)  9 (90) |
| **Equity Considerations for Implementation** | **N (%)** | **N (%)** |
| *Policy document includes equity considerations for implementation for small and/or locally owned restaurants* | N= 0 | N=2 (15.4) |
| Reports using the named equity strategy (Aligns)  Reports NOT using the named equity strategy (Negatively Misaligns) | -  - | 0  2 (100) |
| *Policy document does NOT include equity considerations for implementation for small and/or locally owned restaurants* | N=21 (100) | N=11 (84.6) |
| Reports NOT using an equity strategy (Aligns)  Reports using an equity strategy (Positively Misaligns)  Types of Equity Strategies Reported by Jurisdictions  Additional technical support  Additional time for implementation  Policy communication in multiple language  Financial support  Don’t know | 12 (57.1)  7 (33.3)  5 (71.4)  3 (42.9)  2 (28.6)  1 (14.3)  2 (9.5) | 6 (55.5)  5 (45.5)  4 (80.0)  3 (60.0)  3 (60.0)  3 (60.0)  0 |

^a^ Denominator varies for this item due to missing. N=20 jurisdictions who responded and had a state HDB policy. Italic font indicates findings from review of policy documents. Normal font indicates findings from implementation survey.

HDB: Healthy default beverage.

**Supplemental Table 2:** Alignment of enforcement practices with policy document by jurisdiction level.

|  | **Jurisdictions with State HDB Policy**  **(N=15)** | **Jurisdictions with Local HDB Policy**  **(N= 11)** |
| --- | --- | --- |
| **Enforcement Strategies^b^** | **N (%)** | **N (%)** |
| *Policy document specifies enforcement strategy* | N= 12 (92.3) | N=7 (63.6) |
| Reports using named enforcement strategy (Aligns)  Reports NOT using an enforcement strategy (Negatively Misaligns)  Reports using the INCORRECT enforcement strategy (Negatively Misaligns)  Don’t know | 7 (58.3)  2 (16.7)  2 (16.7)  1 (8.3) | 7 (100)  0  0  0 |
| *Policy document does NOT specify enforcement strategy* | N = 1 (7.7) | N = 4 (36.4) |
| Reports using an enforcement strategy (Positively Misaligns)  Reports NOT using an enforcement strategy (Aligns) | 1 (100)  0 | 1 (25.0)  3 (75.0) |
| **Funding for Enforcement** |  |  |
| *Policy document includes details about enforcement funding* | N=0 | N=0 |
| Reports receiving funding for enforcement (Aligns)  Reports NOT receiving funding for enforcement (Misaligns) | -  - | -  - |
| *Policy document does NOT include details about enforcement funding* | N= 15 (100) | N=11 |
| Reports NOT receiving funding for enforcement (Aligns)  Reports receiving funding for enforcement (Misaligns)  Don’t know | 11 (73.3)  3 (20.0)  1 (6.7) | 4 (36.4)  7 (63.6)  0 |
| **Equity Considerations for Enforcement** | **N (%)** | **N (%)** |
| *Policy document includes equity considerations for enforcement for small and/or locally owned restaurants* | N= 0 (0) | N=0 |
| Reports using the named equity strategy (Aligns)  Reports NOT using the named equity strategy (Negatively Misaligns) | -  - | -  - |
| *Policy document that does NOT include equity considerations for enforcement for small and/or locally owned restaurants* | N=15 (100) | N=11 |
| Reports NOT using an equity strategy (Aligns)  Reports using an equity strategy (Positively Misaligns)  Don’t know  Types of Equity Strategies Reported by Jurisdictions:  Additional time to become compliant | 9 (60.0)  4 (26.7)  2 (13.3)  4 (100) | 11 (100)  0  0  -- |

^b^ Denominator varies for this item due to missing. N=13 jurisdictions who responded and had a state HDB policy.

HDB: Healthy Default Beverage

**Supplemental Table 3**: Free-text responses about healthy default beverage policy implementation and enforcement challenges (n=20)

| **Theme** | **N** | **Example Quote(s)** |
| --- | --- | --- |
| No challenges reported. | 5 | *“Not so far; restaurants have until April 1, 2025 to come into compliance but so far 75 percent are.” –* Jurisdiction with a local policy |
| Limited funding, staffing, and capacity. | 4 | *“There is no additional funding for this...”* – Local jurisdiction implementing a state-level policy.  *“Staffing capacity; the bill was passed right before the COVID-19 pandemic, a difficult time for businesses to implement the law.”* – Local jurisdiction implementing a state-level policy. |
| Implementation challenges among restaurants. | 4 | *“Initially several restaurants started listing ‘drinks’ as the beverage… They thought it was okay since they weren’t explicitly offering sodas, juice, etc.”*  *–* Jurisdiction with a local policy |
| Lack of awareness/knowledge among inspectors. | 3 | *“Sanitarians do not typically study nutrition, so we are enforcing a non-sanitation issue...”* – Local jurisdiction implementing a state-level policy. |
| Limited engagement with health departments and health inspectors. | 2 | *“We have not been able to get support from our Public Health Inspectors which is why we are using a complaint-based approach.”* – Jurisdiction with a local policy |
| Cultural, language, and community-specific barriers | 1 | *“Languages, and addressing different cultural beverages.”* – Local jurisdiction implementing a state-level policy. |
